# Age-associated immune dysregulation and B cell dysfunction drive severe outcomes in SFTSV infection

**DOI:** 10.1371/journal.ppat.1013402

**Published:** 2025-08-12

**Authors:** Eun-Ha Kim, Ho Bin Jang, Se-Mi Kim, Dongbin Park, Young-Il Kim, Seung-Gyu Jang, Suhee Hwang, Woo-Hyun Kwon, Isaac Choi, Jaemoo Kim, Hee-Dong Yang, Hyunjoon Kim, Mark Anthony B. Casel, Min-Suk Song, Jae U. Jung, Young Ki Choi

**Affiliations:** 1 Center for Virus Research Resource, Korea Virus Research Institute, Institute for Basic Science (IBS), Daejeon, Republic of Korea; 2 Center for Study of Emerging and Re-emerging Viruses, Korea Virus Research Institute, Institute for Basic Science (IBS), Daejeon, Republic of Korea; 3 Department of Microbiology, College of Medicine and Medical Research Institute, Chungbuk National University, Cheongju, Republic of Korea; 4 Department of Cancer Biology, Infection Biology Program, and Global Center for Pathogen and Human Health Research, Lerner Research Institute, Cleveland Clinic, Ohio, United States of America; 5 Lead contact; University of New Mexico School of Medicine, UNITED STATES OF AMERICA

## Abstract

Aging significantly influences host immune responses to viral infections, including Severe Fever with Thrombocytopenia Syndrome Virus (SFTSV), which is associated with high mortality in elderly patients. Despite its high fatality rate and pandemic potential, effective therapies remain unavailable, and the age-dependent mechanisms underlying SFTSV pathogenesis are not fully understood. To address this gap, we employed a ferret model (an immunocompetent animal model that mimics human SFTSV infections) and performed multi-tissue single-cell RNA sequencing and histopathological analyses. Our results reveal that, upon SFTSV infection, aged ferrets experience extensive decrease of critical immune cells (particularly B and T cells) due to infection-induced cell death and excessive hemophagocytosis in hematopoietic organs, whereas young-adult ferrets rapidly clear the virus with minimal lymphocyte changes. Notably, aged ferrets display marked immune dysregulation, characterized by non-specific activation of *T-bet* ⁺ age-associated memory B cells (*T-bet*^+^ ABCs) and the proliferation of defective plasmablasts (*MKI67* ⁺ PB1), which serve as major viral reservoirs and drive systemic viral dissemination. Comparative analysis further demonstrated that the *MKI67* ⁺ PB1 subset dominates SFTSV⁺ cells in both aged ferrets and human fatal cases, exhibiting the highest per-cell viral UMI counts. Moreover, monocytes and macrophages in aged ferrets exhibit heightened inflammatory gene expression, contributing to the hyper-inflammatory state observed during infection. Collectively, these insights underscore the critical role of dysregulated memory B cell responses and hyper-inflammation in age-dependent SFTSV pathogenesis, highlighting potential targets for interventions in elderly populations.

## Author summary

SFTSV has emerged as a highly fatal pathogen with no available treatments, promoting its classification by the WHO as a dangerous emerging virus. Although the motality rate among the elderly is particularly high, the age-related dynamics of SFTSV infection remain poorly understood. To address this, our study investigated the impact of aging on SFTSV pathogenesis using a longitudinal single cell RNA-sequencing and histological analysis in ferrets, an animal model that closely mirrors human infection symptoms. The study found significant differences in immune responses between young-adult and aged ferrets. Young-adult ferrets cleared the virus rapidly with stable immune cells, while aged ferrets experienced immune cell decrease, particularly among B and T cells. This decrease was associated with upregulated apoptosis and exhaustion markers, along with excessive hemophagocytosis, leading to immune dysregulation. Notably, aged ferrets showed activation of non-specific age-associated memory B cells, which transitioned into defective plasmablasts. These plasmablasts acted as viral reservoirs with impaired antibody production, facilitating viral spread. Comparative analysis with human data showed similar results, with defective plasmablasts being prevalent in fatal cases. The study provides key insights into age-related vulnerabilities in SFTSV infection, offering perspectives for interventions to protect aging populations.

## Introduction

Severe Fever with Thrombocytopenia Syndrome Virus (SFTSV), a novel *Dabie bandavirus,* is one of the world’s most dangerous pathogens [1], causing fatality rates of up to 27% [2]. Since its emergence in China in 2009 [3], SFTSV has spread across multiple Asian countries, including South Korea, Japan, Pakistan, Myanmar, and Thailand [4–10]. This widespread distribution, combined with its high fatality rate, underscores SFTSV’s pandemic potential and necessitates urgent global attention [1].

Notably, the severity of SFTSV infections exhibits a striking age dependence. More than 90% of clinically diagnosed SFTS patients are 50 years old or older [5], with the highest mortality rates (62.2%) in those aged ≥70 years, followed by rates of 29.7% in those aged 60–69 and 8.1% in those aged 50–59 [11]. This age-associated severity is particularly concerning given the weakening of immune functions in the elderly, known as ‘immunosenescence’ [12]. Moreover, the rapidly growing proportion of individuals over 65 years old, projected to exceed one-sixth of the global population by 2050 [13,14], heightens the risk of severe viral infections in aged populations [15], including SFTSV.

In human cases, SFTSV primarily targets immune cells such as monocytes, macrophages (MΦs), and plasmablasts (PBs), key effectors in antiviral defense, which results in impaired virus-specific humoral immunity and uncontrolled viral replication [16–19]. As the infection progresses, this immune dysregulation can lead to excessive cytokine production and hemophagocytic lymphohistiocytosis (HLH)-like pathology, especially in elderly individuals, ultimately contributing to multi-organ failure and high case-fatality rates [20,21].

Despite the significant impact of age on SFTSV severity, our understanding of the age-related mechanisms underlying the vulnerability of SFTSV remains limited. This knowledge gap is partly due to the challenges in obtaining appropriate clinical samples, particularly invasive tissue samples, from critically ill elderly patients [22,23]. Additionally, most pathological studies on SFTSV-infected patients, regardless of age, have relied on peripheral blood mononuclear cells (PBMC) or post-mortem tissue samples due to the limited accessibility of clinical samples [24–26]. This restriction has impeded comprehensive studies of pathogenesis and hindered progress in developing effective therapies and vaccines for SFTSV.

To address these knowledge gaps, we utilized ferrets (*Mustela putorius furo*), an established immunocompetent model for SFTSV infection [27] that recapitulates age-associated clinical outcomes observed in humans [28]. Several clinical features observed in severe SFTSV patients, such as high fever, thrombocytopenia, leukocytopenia, elevated liver enzymes (AST, ALT), and cytokine storm, are faithfully recapitulated in the aged ferret model [29–32]. Through longitudinal multi-tissue single-cell RNA sequencing (scRNA-seq) and histopathological examinations, we identified an immune signature characterized by defective B cell development that correlates with accelerated disease progression in aged ferrets. Notably, the non-specific activation of *T-bet* ⁺ age-associated memory B cells (*T-bet* ⁺ ABCs) and the expansion of defective *MKI67* ⁺ plasmablasts (PB1) emerged as major viral reservoirs in aged ferrets, resulting in elevated viral loads. Furthermore, myeloid cells including monocytes and MΦs in aged ferrets exhibited significantly increased inflammatory gene expression compared to young-adult ferrets, contributing to the hyper-inflammatory state observed during SFTSV infection. Collectively, our study advances our understanding of age-related immune dynamics in SFTSV infection and provides critical insights for developing targeted interventions in aged populations.

## Results

### Age-dependent dysregulation of immune cell compositions following SFTSV infections

To investigate age-associated differences in SFTSV progression, we infected young-adults (≤2 years; YG) and aged ferrets (≥3 years; AG) with CB1/2014, as previously demonstrated [11,27,33]. While YG showed no mortality and maintained normal platelet counts up to 10 days post infection (dpi), AG ferrets experienced severe fever, thrombocytopenia, and significant weight loss, resulting in 100% mortality by 8 dpi. Only YG ferrets developed serum-neutralizing antibodies at 10 dpi (S1A–S1F Fig).

**Fig 1:**
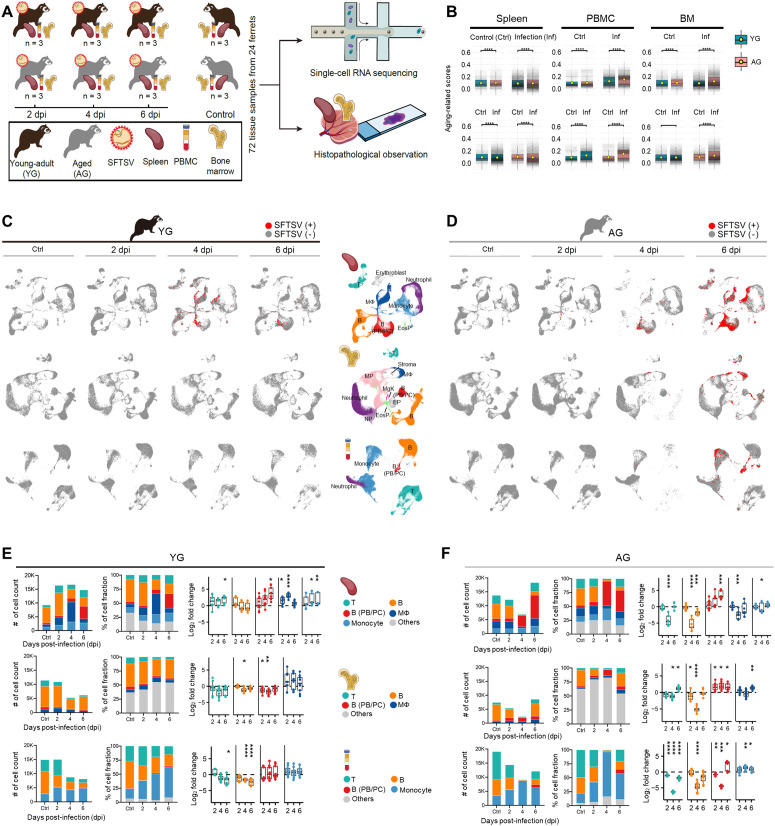
Experimental design and integrated analysis of age-associated dynamics of SFTSV infection. **(A)** Study design for age-associated dynamics of SFTSV infection using ferret animal models. Three tissue samples, including the spleen, peripheral blood mononuclear cell (PBMC), and bone marrow (BM), were taken from young-adult (YG) and aged (AG) ferrets at 2, 4, and 6 days post-infection (dpi), along with control samples. A total of 72 samples from 24 ferrets (YG: n = 12; AG: n = 12) were transcriptionally profiled using droplet-enabled single-cell RNA sequencing (scRNA-seq). In parallel, microscopic analyses of the spleen and BM were performed to validate viral infection dynamics revealed by scRNA-seq. **(B)** Comparison of aging-associated gene expression between YG and AG groups, as well as control (Ctrl) and infection (Inf) samples across three different tissues with individual data points displayed using jittering. **(C, D)** Individual UMAPs depicting the control and infected YG (C) and AG (D) samples. SFTSV^+^ and SFTSV^-^ cells (those with and without SFTSV viral unique molecular identifier [UMI]) are shown in red and gray, respectively. Center, UMAPs of the spleen (top), PBMCs (middle), and BM (bottom) are color-coded by cell types. **(E, F)** Quantification of the number of detected cells, focused on T, B, B (PB/PC), MΦ, monocyte, and other populations, in YG (E) and AG (F). From left, the first and second columns depict the number of cells and the proportion cell types, respectively. The third columns show the log_2_ fold change in each cell-type proportion compared to their control counterparts, respectively. Cell types in the spleen (top), PBMCs (middle), and BM (bottom) are color-coded. Statistical analysis was performed using Wilcoxon rank-sum test with Bonferroni correction (B), Kolmogorov-Smirnov (KS) test (E, F; third column), and multiple Mann-Whiteny test with Bonferroni correction (I). Statistical significance between groups is indicated by asterisks (*P < 0.05, **P < 0.01, ***P < 0.001, ****P < 0.0001). Image in (A) was created with the assistance of BioRender.com.

To explore these age-related different disease outcomes, we performed scRNA-seq and histopathological analyses of the spleen, bone marrow (BM), and PBMC from infected and control ferrets (Fig 1A). A total of 72 tissue samples from 24 ferrets generated 384,951 individual cell transcriptomes. Aging-associated gene-expression scores, calculated for 276 genes [34], were significantly higher in AG ferrets across all tissues (*P* < 0.05, Fig 1B). Interestingly, aging-related gene expression was positively correlated with SFTSV infection burden, even within the same age group, suggesting that infection exacerbates age-associated transcriptional signatures (S1G Fig). Single-cell transcriptome clustering identified 24 cell types across tissues (Figs 1C, 1D, S1H–S1J, and S1 Table). B cell populations are divided into two groups: one composed of PB and plasma cell (PC), labeled as B (PB/PC), and another containing the other B cell types, labeled simply as B. All cell types (24/24) in AG ferrets showed significant increase or decrease during SFTSV infection compared to control, whereas only 46% (11/24) changed in YG ferrets (black asterisks in S1K Fig and S2 Table). The AG group showed a rapid decrease in B and T cells across all tissues by 4 dpi (Fig 1E and 1F), while MΦs and monocytes increased or were maintained over time, except in the spleen at 4 dpi, consistent with direct comparisons of cell proportion changes between YG and AG (red asterisks in S1K Fig). Notably, hematopoietic progenitors such as MgK and EP were significantly decreased in AG ferret BM by 4 dpi, highlighting age-related shifts in immune responses to SFTSV.

### Age-dependent differential B-cell responses during SFTSV infection

**Fig 2:**
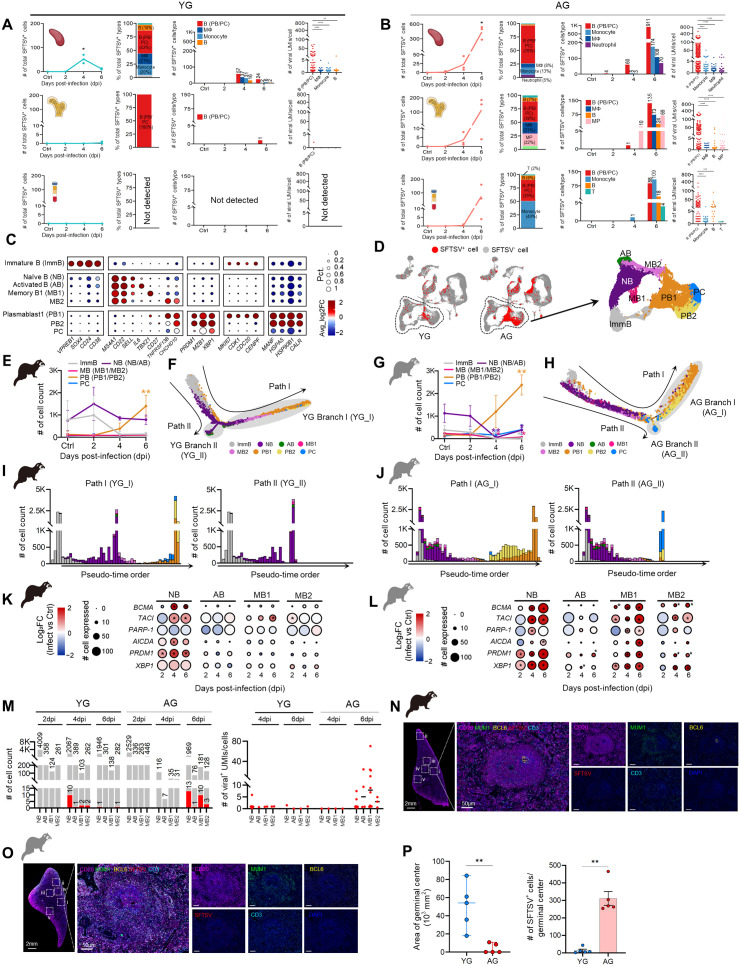
Age-dependent B-cell responses in spleens during SFTSV infection. **(A, B)** Quantification of the number of SFTSV^+^ cells, focused on top 4 highly infected cell types, in YG (A) and AG (B). Columns show total SFTSV^+^ cells, percentage fraction, cell counts during infection, and per-cell viral UMI in each type. **(C)** Dotplot showing the percentage and average expression of DEGs associated with canonical markers and cell cycle/differentiation. **(D)** Left: UMAPs of SFTSV^+^ (red) and SFTSV^-^ cells (gray) in YG and AG spleens, with dotted lines indicating B cell populations. Right: Enlarged B cell UMAP regions are colored by subsets. **(E-H)** B cell subset counts in controls and each dpi from infected YG (E) and AG (G). Trajectories, colored by subsets, show developmental paths and branches in YG (F) and AG (H). **(I, J)** Pseudo-time plots of cell counts with colors representing distinct B cell subsets in YG (I) and AG (J). **(K, L)** Dot plots of average gene expression for subsets vs. controls (color) and target-expressing cell counts (circle size) in YG (K) and AG (L). **(M)** Left: Total (gray) and SFTSV^+^ cells (red) in the splenic naïve B (NB), activated B (AB), memory B1 (MB1; *T-bet*^+^ ABCs), and MB2 cells during infection between YG and AG. Right: Per-cell viral UMI counts in NB, AB, MB1, and MB2 populations from the infected groups in YG and AG. **(N, O)** Selected spleen ROIs (i-v) at 4 dpi from YG (N) and AG (O) show cross-sections, enlarged ROI i, and stained images of CD20^+^ B (magenta), MUM1^+^ PB (green), BCL6^+^ germinal centers (yellow), SFTSV (red), CD3^+^ T (cyan), and DAPI (blue). Scale bars: 2 mm (left), 50 µm (middle). **(P)** Germinal center area (10^3^ mm^2^) and SFTSV^+^ counts in sectioned spleen images from YG (blue) and AG (red). Groups were compared using Wilcoxon rank-sum test (K, L), and two-tailed Mann-Whitney U test (A, B, E, F, G, H, and P) with Bonferroni correction, respectively. Statistical significance is indicated by asterisks (*P < 0.05, **P < 0.01, ***P < 0.001, ****P < 0.0001). Significance of (K) and (L) are represented with an asterisk (*) due to space limits (all *P* < 0.05).

Next, we identified and quantified SFTSV-infected (SFTSV^+^) cells across the tissues (S3 Table). In YG group, SFTSV^+^ cells in the spleen peaked at 4 dpi, then declined by 6 dpi, while AG ferrets exhibited earlier as 2 dpi and significantly increased up to 6 dpi (Fig 2A and 2B; left panels). Consistent, real-time reverse transcription polymerase chain reaction (qRT-PCR) and RNAscope confirmed transient viral replication in YG, contrasting with sustained viral replication in AG ferrets (S2A and S2B Fig). Among cell types, SFTSV^+^ cells were predominantly B (PB/PC) cells (59.4%), followed by monocytes (15.2%), M*Φ* (10.3%), B cells (4.1%), myeloid progenitors (MP) (3.7%), neutrophils (3.6%), T cells (2.2%), and/or others in both groups (Fig 2A and 2B; middle panels). Notably, viral UMI counts per cell (SFTSV unique molecular identifiers [UMIs]/cell) increased approximately 30-fold in B (PB/PC) cells compared to other SFTSV^+^ cells across three tissues (Fig 2A and 2B; right panels). Further, AG B (PB/PC) cells exhibited twice the UMIs per cell compared to their YG counterparts (S2C–S2F Fig).

To further investigate B cell behavior during infection, we categorized B cell lineages into subsets: immature B (ImmB), naïve B (NB), activated B (AB), memory B (MB1-2), plasmablast (PB1-2), and plasma cell based on gene markers, visualized on uniform manifold approximation and projection (UMAP) (Fig 2C and 2D) [35]. In YG ferrets, NB cells increased at 2 dpi, returning to baseline by 4 dpi, which correlated with a rise in PBs without notable changes in MB populations (Figs 2E and S2G). In contrast, AG ferrets showed a marked decline in both NB and MB cells, particularly MB1, by 4 dpi, followed by a sharp increase in PBs from 4 to 6 dpi, doubling PB numbers, especially PB1, relative to YG (Figs 2G and S2H). Trajectory analyses revealed that, in YG ferrets, NBs primarily differentiated into PB/PCs (Branch I, YG_I) through activation of transcription factors (TFs; *PRDM1* and *XBP1*) and into a mixed AB/MBs (Branch II, YG_II), characterized by *IL6*, *BANK1*, and *CCR6* expression (Figs 2F, 2I, S2I, and S2K). In AG ferrets, however, NBs and MBs simultaneously differentiated into a PB-dominant branch (Branch I, AG_I) and a mixed PB/PC branch (Branch II, AG_II) (Figs 2H, 2J, S2J, and S2L). Interestingly, among the MB subsets, MB1 cells expressed canonical differentially expressed genes (DEGs) linked to *T-bet*^+^ ABCs (S2M Fig) [36,37]. Notably, these AG MB1 showed earlier and more robust expression of TFs involved in PB/PC differentiation [38,39] starting from 2 dpi, suggesting both AG NB and MB1 subsets early differentiation into PB/PC subsets (Figs 2K, 2L, and S2N). Additionally, AG MB1 cells exhibited a higher proportion of SFTSV⁺ cells compared to YG [AG: 10%, YG: 2%], with the highest viral UMI/cell values among B cell subsets, 12.3-fold higher than their YG counterparts [AG: 8.76, YG: 0.71] (Figs 2M, S2O). However, this difference did not reach statistical significance across biological replicates. To further validate these observations, we performed flow cytometry and qRT-PCR analysis. FACS analysis revealed a significantly higher frequency of CD11c⁺ T-bet⁺ NP⁺ MB1 cells in AG ferrets compared to YG, indicating elevated levels of viral infection (S2P Fig, left and middle). Consistently, qRT-PCR quantification of viral RNA in sorted MB1 cells showed persistently higher viral loads in AG ferrets across multiple time points (2, 4, and 6 dpi) (S2P Fig, right), corroborating the scRNA-seq–based observations.

Gene-expression analysis further revealed delayed activation-induced cytidine deaminase (*AICDA*) expression, a gene critical for antibody maturation via somatic hypermutation and class switch recombination [40–42], in AG NBs. *AICDA* was significantly upregulated in YG NBs by 4 dpi but remained limited in AG NBs until 6 dpi. Meanwhile, in AG ferrets, *AICDA* expression markedly increased in MB1 cells at 4 dpi, unlike in YG (Figs 2K, 2L, and S2N). Interestingly, this increase was accompanied by the elevated expression of *PARP-1*, which inhibits AICDA activity. Moreover, the expression of *BCMA* and *TACI*, key receptors for BAFF/APRIL that promote B cell activation and differentiation independent of antigen stimulation, was significantly upregulated from as early as 2 dpi. Consistent with these findings, qRT-PCR analysis at 6 dpi further confirmed elevated *BCMA* and *TACI* expression in MB1 cells, mirroring the expression patterns observed in the scRNA-seq dataset (S2Q Fig). These findings collectively suggest that MB1 cells undergo non-specific activation in response to SFTSV infection.

Given the prominent expansion of T-bet^+^ ABC-like cells in AG ferrets during SFTSV infection, we further investigated potential upstream signaling pathways involved in their activation. qRT-PCR analysis of spleen tissues revealed no statistically significant differences in the expression of *TLR7* or *MyD88* between YG and AG ferrets. In contrast, AG ferrets showed significantly elevated expression of *STAT4* and *PPP3CB*, key components of the IL-12/STAT4 signaling axis, whereas no such increase was observed in YG ferrets. These findings suggest that cytokine-mediated pathways, particularly IL-12/STAT4 signaling, may play a dominant role in the aberrant activation of T-bet^+^ ABCs in aged hosts. Consistently, comparative expression profiling of genes involved in T-bet^+^ cell regulation further supported the involvement of extrinsic cytokine signaling in driving age-associated ABC activation (S2R Fig). Microscopic examination of virus-infected spleens revealed significant structural differences in germinal centers (GC): YG spleens displayed distinct BCL6^+^ germinal centers with a reduced presence of SFTSVs, while AG spleens exhibited impaired germinal center formation, with abundant SFTSVs, which likely compromised immune responses (Fig 2N and 2O). The quantification of the germinal center area in each Region of Interest (ROI) and the number of SFTSV^+^ cells within each geminal center highlight the under-developed state and the significant presence of SFTSV^+^ cells in the germinal centers of AG ferrets (Fig 2P).

### Defective maturation and prolonged proliferation of plasmablast populations in SFTSV-infected aged group

**Fig 3:**
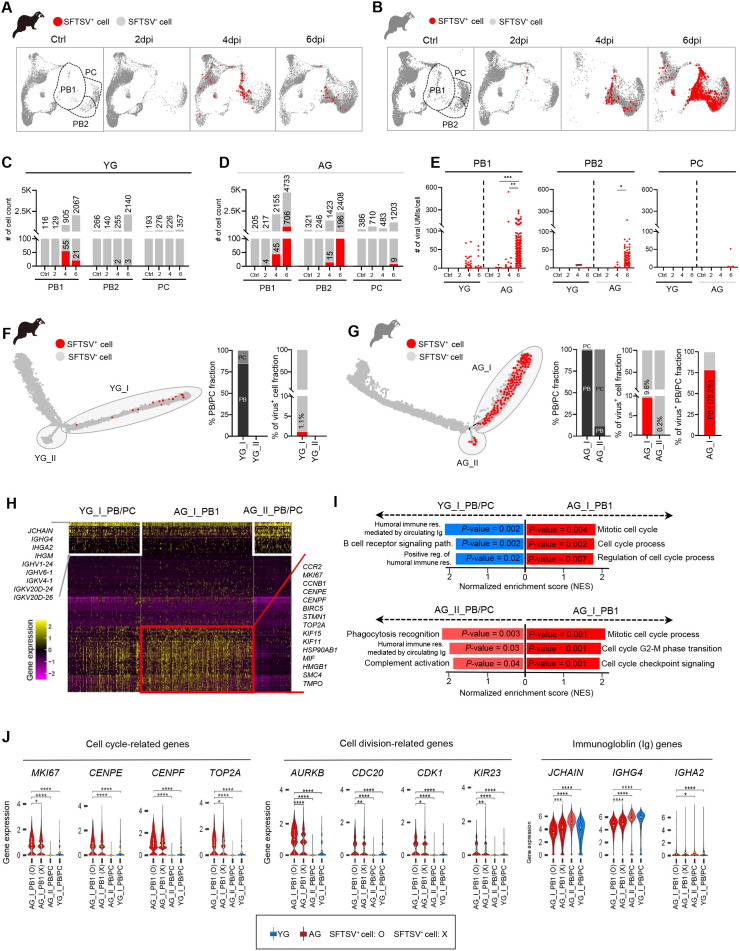
Distinctive transcriptional changes in plasmablast populations of the aged group during SFTSV infection. **(A, B)** UMAPs show splenic PB and PC populations in YG (A) and AG (B) at control, 2, 4, and 6 dpi. PB/PC subsets are marked by dotted lines; SFTSV^+^ and SFTSV^-^ cells are red and gray. Subset(s) of PB and PC are marked by dotted lines. **(C, D)** Quantification of the number of PB1, PB2 and PC in YG(C) and AG (D). Gray and red bars represent Total and SFTSV^+^ cells. **(E)** Comparisons of the number of viral UMI/cells detected within the PB1, PB2, and PC1 subsets in YG and AG during infections. **(F, G)** YG (F) and AG (G) trajectories highlight PB/PC-containing branches (solid circles: YG_I/II; AG_I/II) with SFTSV^+^ (red) and SFTSV^-^ (gray) cells. Percentage fractions of PB, PC, and SFTSV^+^ PB/PC populations in YG_I/II and AG_I/II are shown. **(H)** Heatmap displaying DEG expression in PB and PC populations belonging to YG_I, AG_II, and PB1 belonging to AG_I branches. Selected DEGs associated with B cell immune function (left) and cell cycle/differentiation (right) are shown. **(I)** Gene set enrichment analysis highlighting highly enriched GO biological pathways between AG_I_PB1 vs. YG_I_PB/PC (top) and AG_I_PB1 vs. AG_II_PB/PC (bottom), with nominal *P*-values and normalized enrichment scores (NES). **(J)** Violin plots showing the expression of the cell-cycle, cell division, and Ig-associated genes in AG_I_PB1, AG_II_PB/PC, and YG_I_PB/PC subsets, separated by virus infection. Statistical significance is indicated above the respective plots. Groups were compared using Bonferroni-adjusted multiple comparisons using the Mann-Whitney test (E) and Bonferroni-adjusted Wilcoxon rank-sum test (J), respectively. Statistical significance between groups is indicated by asterisks (*P < 0.05, **P < 0.01, ***P < 0.001, ****P < 0.0001).

To investigate the age-related differences in B cell transitions, we analyzed transcriptional changes in PB and PC populations. AG ferrets displayed a significant (>2-fold) expansion in PBs, particularly in the *MKI67*^+^ PB1 subset, compared to YG ferrets (Figs 3A, 3B, and S3A). *MKI67*^+^ PB1 cells in AG ferrets were notably susceptible to SFTSV infection, showing the highest number of SFTSV^+^ cells and viral UMIs/cell (Fig 3C–3E). Trajectory analyses revealed distinct patterns of SFTSV^+^ PB/PC differentiation between age groups. In YG ferrets, SFTSV^+^ PB/PCs were confined to a single trajectory branch (YG_I). In contrast, in AG, SFTSV^+^ PB/PCs diverged into two branches: AG_I (predominantly PB populations) and AG_II (a mix of PBs and PCs) (Figs 3F, 3G, and S3B). The proportion of SFTSV^+^ PB/PCs was markedly higher in AG_I (9.6%) compared to YG_I (1.1%), with *MKI67*^+^ PB1 cells constituting 78.2% of SFTSV+ cells in AG_I.

Transcriptional analysis further revealed functional differences between these populations. In contrast to the PB/PCs in YG_I and AG_II, the PB1 cells in AG_I showed enrichment of cell cycle-related genes (e.g., *MKI67*, *CENPE*, *CENPF*, and *TOP2A*) but downregulated immunoglobulin (Ig) gene expression (e.g., *JCHAIN*, *IGHG4*, IGKV4, and *IGHV*), suggesting impaired maturation into functional PCs (Figs 3H, 3I, S3C, and S3D). Ingenuity Pathway Analysis (IPA) supported these findings, indicating the downregulation of G2/M DNA damage checkpoint regulation in AG_I_PB1 cells, consistent with enhanced cell division (S3E and S3F Fig). Moreover, cell cycle-related genes and cell division-related genes (e.g., *AURKB*, *CDC20*, and *KIF23*) were most upregulated in SFTSV^+^ PB1 cells within AG_I, while Ig genes were downregulated (Fig 3J). Consistently, additional qRT-PCR analysis confirmed significantly higher expression of key cell cycle and division-related genes (MKI67, CENPF, AURKB, and CDC20) in PB1 cells from aged ferrets compared to young ferrets, reinforcing our conclusions from scRNA-seq data. (S3G Fig). This pattern highlights a phenotype of continuous proliferation without immune maturation.

To validate these transcriptomic findings, we performed FACS analysis to identify Ki-67^+^ CD38^+^ CD79a^+^ PB1 cells and quantified NP^+^ cell frequency within this subset. In line with the scRNA-seq results, AG ferrets showed a significantly higher proportion of NP^+^ PB1 cells, accompanied by markedly elevated viral RNA levels as determined by qRT-PCR (S3H Fig). To complement these analyses, we instead assessed plasmablast function by examining surface expression of immunoglobulin isotypes (IgG, IgM, and IgA) on CD38^+^ CD27^+^ CD79a^+^ cells (plasmablast), providing insight into their antibody-producing potential in ferrets. YG ferrets displayed higher and more dynamic Ig expression following infection, whereas AG ferrets exhibited a decline in Ig levels post-infection despite elevated baseline expression (S3I Fig). Collectively, these findings collectively suggest that in AG ferrets, *MKI67*^+^ PB1 cells exhibit dysregulated expansion and impaired function, which serve as a source of viral replication without effective immune responses during SFTSV infection.

### Aged myeloid cells act as amplifiers of chronic inflammatory responses rather than virus reservoirs

**Fig 4:**
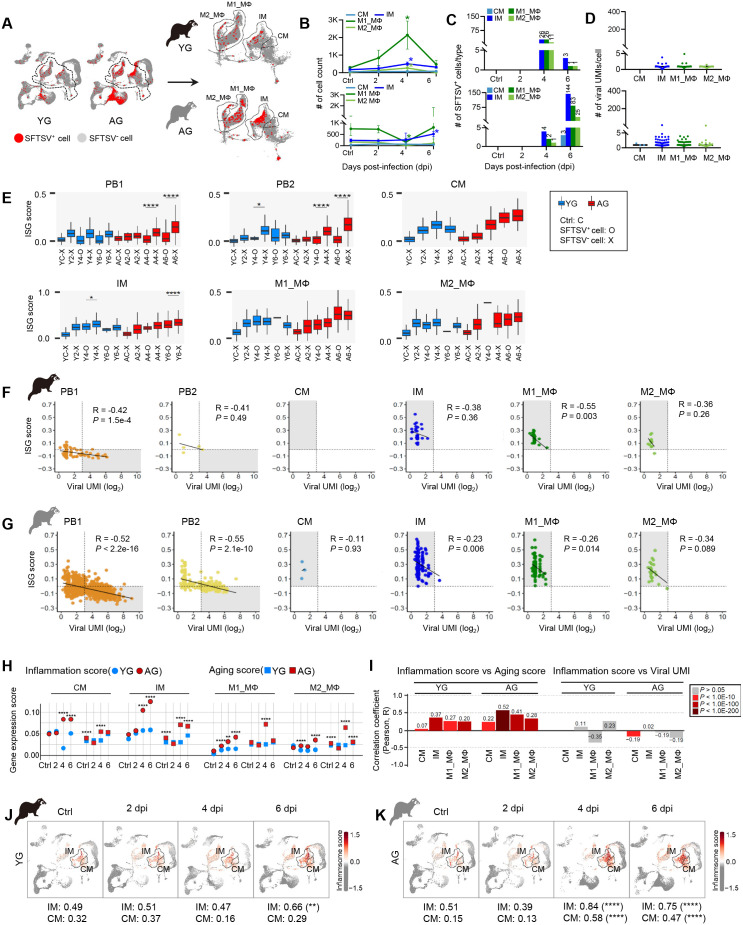
Transcriptomic characterization of splenic myeloid cell populations. **(A)** UMAPs representing SFTSV^+^ (red) and SFTSV^-^ (gray) cells in YG and AG spleen with monocytes and MΦ populations (CM, IM, M1_MΦ, M2_MΦ) highlighted by dotted lines. **(B)** Population changes in the CM, IM, M1_MΦ, and M2_MΦ subsets between YG (top) and AG (bottom) over dpi. Asterisks indicate statistically significant differences from controls. **(C)** SFTSV^+^ cell quantification in CM, IM, M1_MΦ, M2_MΦ subsets for YG (top) and AG (bottom). Total SFTSV^+^ counts are noted above bars. **(D)** The number of SFTSV viral UMI/cell detected within CM, IM, M1_MΦ, M2_MΦ in YG (top) and AG (bottom). **(E)** ISG scores in PB1, PB2, CM, IM, M1_MΦ, M2_MΦ subsets in YG (blue) and AG (red) at control (C) and dpi (2, 4, 6). SFTSV^+^ (O) and SFTSV^-^ (X) cells are distinguished. Asterisks show significant differences. **(F, G)** Correlation analysis of ISG score and viral UMI (log_2_) in PB1, PB2, CM, IM, M1_MΦ, and M2_MΦ of YG (F) and AG (G). Each dot represents each cell, with a trend line illustrating the overall pattern. Pearson correlation coefficient (R) and *P*-values are shown. **(H)** Inflammation scores (circle) and aging scores (square) in CM, IM, M1_MΦ, M2_MΦ subsets of YG (blue) and AG (red) across controls and dpi (2, 4, 6). **(I)** Correlation of inflammation scores with aging scores (left) and viral UMI counts (right). Bar charts show Pearson R, colored by *P*-values. **(J, K)** UMAPs of inflammasome scores in IM and CM subsets in YG (J) and AG (K) at control and dpi. Mean inflammasome scores are indicated by color intensity. Statistical comparisons were performed with two-tailed Mann-Whitney U (B), and Wilcoxon rank-sum test (E, H, J, and K) corrected by Bonferroni method, respectively. Statistical significance between groups is indicated by asterisks (*P < 0.05, **P < 0.01, ***P < 0.001, ****P < 0.0001).

We next examined the role of splenic monocyte and MΦ cells, which exhibited the second and third-highest frequencies of SFTSV^+^ cells across tissues (Figs 2A, 2B, and 4A). These myeloid cells were classified into classical (CM), intermediate monocytes (IM), and M1_MΦ and M2_MΦ based on canonical marker DEGs (S4A Fig). Quantitative analyses revealed a significant increase of YG M1_MΦ populations at 4 dpi, while AG M1_MΦ populations sharply decreased with a rise in apoptosis-related genes, suggesting age-associated differences in cell dynamics and apoptosis (Figs 4B and S4B). Similar to PBs, SFTSV^+^ myeloid cells in YG spleen surged at 4 dpi but decreased by 6 dpi, while AG groups dramatically increased SFTSV^+^ cells until 6 dpi (Fig 4C). Despite these increases, viral UMI/cell counts remained low across all myeloid cell subsets, suggesting that these cells are not primary sites of viral replication (Fig 4D). Analysis of Type I interferon-stimulated gene (ISG) expression scores across myeloid subsets and PBs revealed that AG PB1 cells exhibited significant ISG downregulation in SFTSV^+^ cells, showing a strong negative correlation between ISG scores and viral UMIs/cell. In contrast, myeloid subsets displayed a progressive increase in ISG expression over time in both virus-infected and bystander cells, independent of infection status, and showed weak or no correlation between ISG scores and viral UMIs/cell (Fig 4E–4G). These finding further support the hypothesis that PB1 cells, rather than myeloid cells, serve as primary viral reservoirs in AG ferrets.

Notably, AG myeloid cells exhibited higher scores for inflammatory-related genes and aging-related genes [34] compared to YG (Fig 4H). Correlations among aging, inflammation, and viral infection revealed that inflammation scores in monocytes and MΦs increased with aging scores, though only weakly correlated with viral UMI levels (Fig 4I). Notably, inflammasome scores (based on *IL1B*, *IL18*, *NLRP3*, *GSDMD*, *PYCARD*) [43] remained stable in YG CMs and IMs during infection but increased consistently in AG subsets from 4 to 6 dpi (Fig 4J and 4K). Consistent with our findings, qRT-PCR analysis confirmed elevated IL-1β and IL-18 expression in aged myeloid cells following SFTSV infection, indicating an age-associated enhancement of inflammatory responses (S4C Fig). This aligns with previous studies suggesting that proinflammatory cytokines can promote viral replication and amplify tissue inflammation, thereby increasing susceptibility and disease severity in aged populations [21,44]. These results suggest higher and prolonged inflammation in AG ferrets driven by aging-associated monocyte and MΦ responses.

### Comparative analysis of aging-related inflammation and viral infection in BM and PBMCs

**Fig 5:**
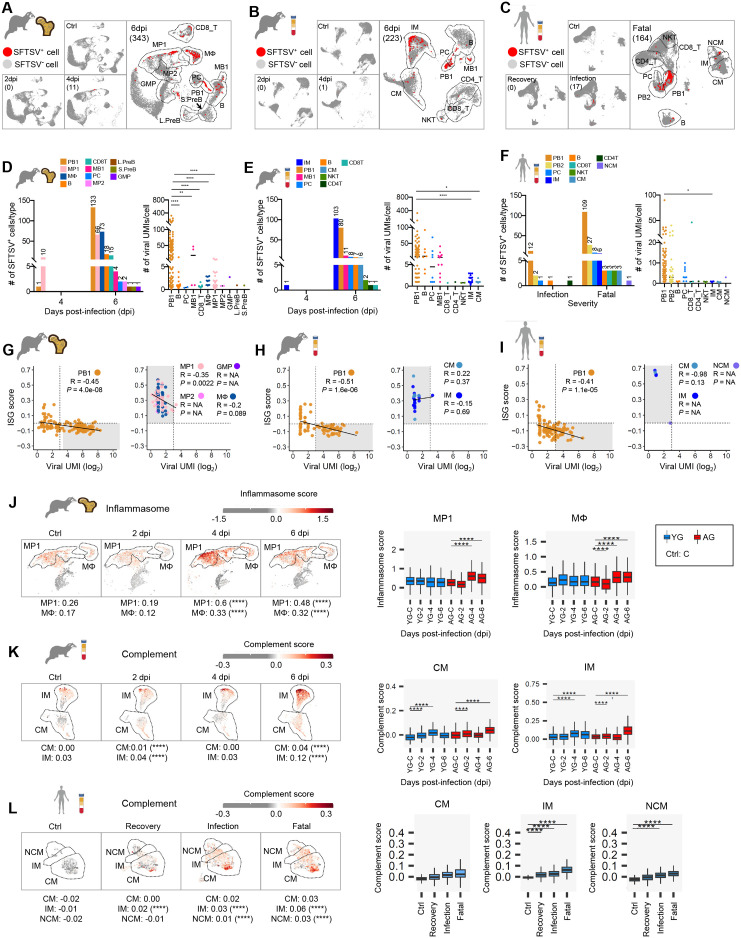
Comparisons of infection dynamics between ferrets and humans. **(A-C)** UMAPs of SFTSV^+^ (red) and SFTSV^-^ (gray) cells in AG BM (A), AG PBMC (B), and human PBMC samples (C) across infection statuses (Control, Infection, Recovery, Fatal). Total SFTSV^+^ cell counts are noted. Subclusters belonging to B, B (PB/PC), T (CD4, CD8), Monocyte, and MΦ are highlighted by dotted lines. **(D-F)** Bar graphs show SFTSV^+^ cell counts per subset in AG BM (D), AG PBMC (E), and human PBMC (F) at 4, 6 dpi, and patient status. Scatter plots on the right show viral UMIs/cell across immune cell types with significance thresholds. **(G-I)** Scatter plots of viral UMIs (Log_2_) and ISG scores in PB1 and selected myeloid subsets from AG BM (G), AG PBMC (H), and fatal human PBMC (I). Each dot is a cell; trend lines illustrate correlations. Pearson R and *P*-values are noted. **(J-L)** UMAPs of inflammasome scores in MP1 and MΦ (AG BM, J) and complement scores in CM and IM (AG PBMC, K), and CM, IM, NCM (human PBMC, L). Boxplots summarize inflammasome/complement scores across conditions (Ctrl, 2, 4, 6 dpi in YG, AG; Control, Recovery, Infection, Fatal). Statistical analysis was performed using Bonferroni-adjusted multiple Mann-Whitney test (D-F) and Bonferroni-adjusted Wilcoxon rank-sum test (J-L), respectively. Statistical significance between groups is indicated by asterisks (*P < 0.05, **P < 0.01, ***P < 0.001, ****P < 0.0001).

To assess systemic SFTSV progression, we analyzed BM and PBMC samples from both ferret groups and conducted a comparative study with human PBMCs at various SFTSV infection stages (recovery, infection, fatal cases) and controls (Figs 5A–5C and S5A–S5D) [18,19]. Integrating scRNA-seq data from human PBMCs (n = 175,762 cells), we identified 13 subsets. Interestingly, human PBMCs showed increasing SFTSV^+^ cells with disease severity (n = 0, 17, and 164 in recovery, infection, and fatal cases, respectively, indicating a relationship between viral loads and SFTSV severity.

B cells and myeloid populations comprised the majority (~64–94%) of SFTSV^+^ cells in both AG ferrets and severe patients, with *MKI67*^+^ PB1 cells showing the highest viral UMI count/cell (Figs 5D–5F and S5E). Among monocytes, IMs displayed the highest number of infected cells and the higher viral UMI count/cell than other monocytes in both ferret and human PBMCs, though viral UMIs remained below 5. Notably, both ferrets and humans exhibited a significant negative correlation between ISG scores and viral UMIs in *MKI67*^+^ PB1 cells, but this relationship was absent in myeloid subsets, which maintained elevated ISG scores irrespective of viral load (Figs 5G–5I and S5F–S5H). Similar to splenic myeloid cells, inflammation scores in MP1, MΦ, CM, and IM from BM and PBMCs in AG ferrets were consistently higher compared to YG at 4 and 6 dpi (S5I and S5J Fig). In particular, inflammasome-related inflammation scores significantly increased in MP1 and MΦ from AG ferret BM between 4 and 6 dpi, while remaining largely unchanged in YG (Fig 5J). Interestingly, PBMCs from AG ferrets displayed elevated complement scores, reflecting transcriptional profiles similar to those observed in fatal human SFTSV infections (Fig 5K and 5L) [19].

### Excessive hemophagocytosis-driven immune cell depletion in aged ferrets

**Fig 6:**
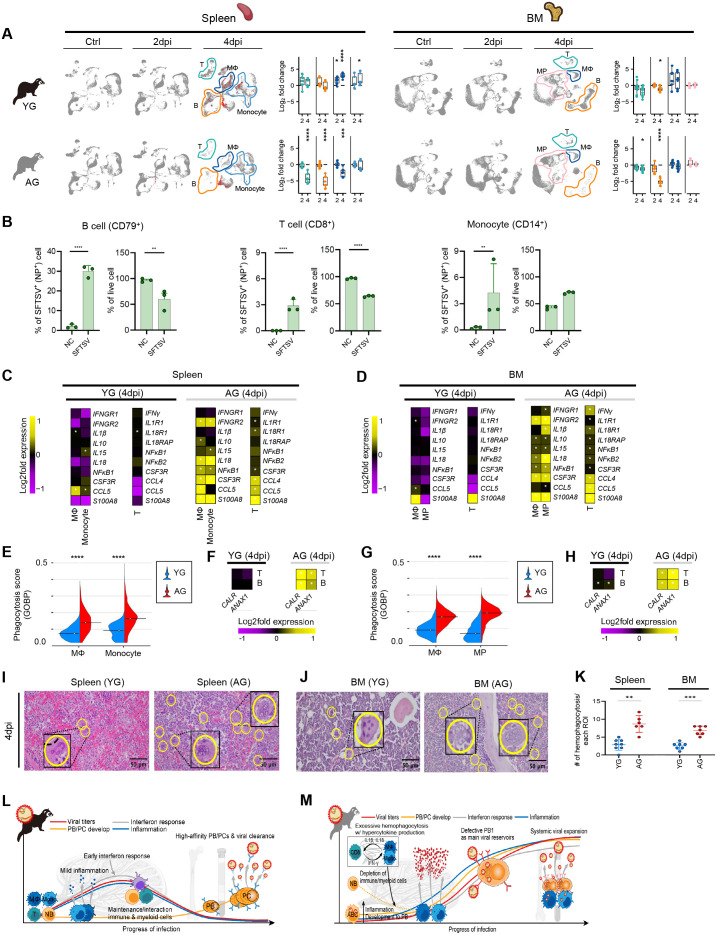
Gene expression associated with functional immune responses in the spleen and BM across both age groups at 4dpi. **(A)** Left: UMAPs show spleen and BM in YG (top) and AG (bottom) at control, 2, and 4dpi. B, T, MΦ, monocyte, and MP subsets are marked by dotted lines; SFTSV^+^ and SFTSV^-^ cells are colored as red and gray, respectively. Right: Boxplots show the log_2_ fold change in each cell type’s proportion compared to their control counterparts from YG (top) and AG (bottom). **(B)** The percentages of SFTSV NP⁺ cells and live cells among B cells (CD79⁺), T cells (CD8⁺), and Monocytes (CD14^+^) are shown under normal conditions (NC) and after SFTSV infection (SFTSV). **(C, D)** Heatmaps showing log_2_ fold changes in the expression of key hemophagocytosis-related genes in splenic (spleen, C) and bone marrow (BM, D) from YG and AG ferrets at 4 dpi during SFTSV infection. **(E, G)** Split violin plots displaying the phagocytosis scores at 4 dpi in two tissues: the spleen (E) and BM (G) for MΦ and monocytes in the spleen, and MΦ and monocyte precursors (MP) in the BM. Data are presented for YG (blue) and AG (red) ferret. **(F, H)** Heatmaps showing the log_2_ fold expression of “eat-me” signals, *Calreticulin* (*CALR*) and *ANXA1*, in B and T cell types from the spleen (F) and in BM (H) of YG and AG at 4 dpi with SFTSV. **(I, J)** Microscopic image of the spleen (I) and BM (J), showing hemophagocytosis in YG and AG ferrets at 4 dpi (H&E, 400X magnification). **(K)** The number of hemophagocytosis events per each region of interest (ROI) in the spleen and BM of YG and AG groups. **(L, M)** Schematic presentation of the difference of immune response between YG (L) and AG (M) ferrets during SFTSV infection. SFTSV infection in AG ferrets caused significant B and T cell depletion, driven by cell death, hemophagocytosis, and hypercytokinemia, unlike in YG ferrets. Additionally, non-functional PB1 populations from T-bet + ABCs acted as viral reservoirs, leading to higher viral loads, hyperinflammation, and systemic viral spread. Statistical significance was assessed using the Wilcoxon rank-sum test (A, C, D, E, F, G, and H) with *P* values less than 0.05 indicated by an asterisk (*) due to space constraints (C, D, F, and H). Additionally, a two-tailed Mann-Whitney U test was employed (B, K). Bonferroni method was conducted for multiple testing correction. Significant differences between groups are denoted by asterisks (*P < 0.05, **P < 0.01, ***P < 0.001, ****P < 0.0001). Images in (L, M) were created with the assistance of BioRender.com.

SFTSV infection in AG ferrets induced striking alterations in immune cell populations, particularly from 4 dpi, contributing to systemic viral expansion. To investigate the mechanisms underlying age-specific immune cell decrease, we analyzed transcriptomes of B, T, and myeloid cells in spleen and BM, where pronounced cell decreases and SFTSV+ cell frequency were observed (Fig 6A).

In AG ferrets, most immune cells, particularly B and T cells, showed marked decrease and upregulated expression of apoptosis-related genes (*FAS*, *ANXA5*), necroptosis (*CASP8*, *RIPK1*), and pyroptosis (*GSDMD*, *NLRP3*) [43,45,46], in contrast to YG ferrets (S6A, S6B Fig). To investigate age-associated alterations in T cell function during SFTSV infection, we examined both exhaustion markers and antiviral cytokine responses. Transcriptomic and protein-level analyses revealed that *PDCD1* (encoding PD-1) was significantly upregulated in CD4^+^ T cells from AG ferrets at baseline and during infection, indicating a pre-existing exhaustion phenotype (S6C Fig). In contrast, *HAVCR2* (TIM-3) expression did not differ significantly between age groups. To assess whether these cell death mechanisms were directly associated with virus infections, in vitro infection studies were conducted with naïve splenocytes. Following 1-hour viral exposure, cells were cultured for 5 days, and samples were collected daily from day 2–5 post-infection to measure nucleoprotein (NP)-positive cells and assess viability across immune subsets. Interestingly, B and T cells were highly susceptible to SFTSV infection, with their populations declining rapidly by 4 dpi (Fig 6B). Although myeloid cells were also susceptible to infection, their populations remained relatively stable, suggesting a distinct mechanism of immune cell decrease. Notably, myeloid cells in AG ferrets showed significantly elevated expression of inflammatory cytokines, including *IL-1β, IL-10, IL-18, NF*κ*B1*, and *S100A8*, as well as upregulated *IFNGR2* expression throughout infection, especially 4dpi (Figs 6C, 6D, S6D, and S6E). Concurrently, T cells exhibited enhanced expression of IFN-γ and IL-18 receptor genes (*IL18R1* and *IL18RAP*), pointing to heightened cross-activation between T and myeloid cells [47]. Functionally, ELISpot assays showed comparable IFN-γ secretion in both YG and AG ferrets post-infection, whereas IL-2 secretion was significantly higher in AG ferrets at 6 dpi (p < 0.01). Intracellular cytokine staining revealed that AG ferrets exhibited enhanced IFN-γ expression in CD4 ⁺ T cells, while YG ferrets mounted stronger IFN-γ responses in CD8 ⁺ T cells. These findings suggest that aged ferrets display altered T cell activation patterns characterized by increased exhaustion, particularly within the CD4 ⁺ compartment, and skewed cytokine responses, which may contribute to impaired antiviral immunity (S6D–S6F Fig). By 4 dpi, myeloid cells in AG ferrets demonstrated significantly higher phagocytosis scores than their YG counterparts (Figs 6E, 6G, S6G, and S6H). Notably, “eat-me” signals [48] such as *Calreticulin* (*CALR*) and *ANXA1* were upregulated in multiple AG cell types, including B and T cells in the spleen and BM (Figs 6F, 6H, S6I, and S6J). These signals correlated with heightened phagocytic activity by myeloid cells and likely contributed to the depletion of immune cells via hemophagocytosis [49]. Histopathological analyses confirmed increased hemophagocytosis in the spleen and BM of AG ferrets from 4 to 6 dpi (Figs 6I–6K and S6K–S6M), consistent with the transcriptional data. These findings indicate that immune cell decrease in AG ferrets results not only from infection-induced cell death mechanisms but also from an inflammatory cytokine-driven increase in hemophagocytosis, particularly within hematopoietic organs. This dual mechanism significantly exacerbates the pathological impact of SFTSV infection.

In summary, SFTSV-infected AG ferrets exhibited early and profound immune cell decrease, including T and B cells, driven by a combination of direct cell death and hemophagocytosis associated with hypercytokinemia, in contrast to their YG counterparts. Concurrently, non-functional *MKI67*^+^ PB1 populations were rapidly derived from *T-bet*^+^ ABCs, acting as major viral reservoirs while failing to produce virus-specific antibodies. This dysregulated immune response contributed to increased viral loads, heightened inflammation, and systemic viral dissemination (Fig 6L and 6M).

## Discussion

Severe outcomes in aged SFTS patients are likely driven by a convergence of immunological deficits that accompany aging, including rapid lymphocyte depletion, impaired viral control within B cell compartments, and heightened inflammatory responses. In this study, our aged ferret model faithfully recapitulated these clinical features, offering mechanistic insights into how age-associated immune dysregulation may compromise host resilience and promote disease severity during SFTSV infection.

Our longitudinal multi-tissue scRNA-seq analysis revealed that SFTSV infection not only elevated aging-related gene expression but also contributed to a pronounced loss of immune cells, particularly B and T cells, in aged ferrets, along with heightened myeloid-driven inflammation. This immune dysregulation appears to be influenced by aging-related processes, such as inflammaging [50], which persistently activates cytokine storm-related genes. Early T cell decrease was linked with excessive expression of infection-induced cell death genes associated with apoptosis, necroptosis, and pyroptosis, contributing to impaired viral clearance and facilitating systemic infection during the initial stages. This parallels the immune collapse observed in severe human SFTS cases [46]. Furthermore, excessive hemophagocytosis in the BM and spleen of AG ferrets, combined with elevated cytokine levels, reflects features of HLH, exacerbating immune cell decrease during the early phase of SFTSV infection [51].

scRNA-seq analysis of splenic B cells revealed that age-related severity may stem from compromised early B cell responses. In AG ferrets, immature and naïve B cells rapidly declined following infection, in contrast to YG ferrets, where these populations were maintained. Instead, non-specific memory B cells, particularly *T-bet*^+^ ABCs, served as the source of defective plasmablasts (*MKI67*^+^ PB1). These findings align with studies showing *T-bet*^+^ ABCs play a role in plasmablast differentiation during viral infections, e.g., SARS-CoV-2 [52], but are also implicated in inflammaging [36]. Notably, unlike YG PBs, most SFTSV-infected PB populations in AG ferrets exhibited downregulation of B cell-related immune maturation genes but upregulation of cell cycle-related genes. AG PB subsets, particularly SFTSV^+^ *MKI67*^+^ PB1 cells, showed inactivation of the G2/M checkpoint and enhanced expression of cell division-related genes, alongside delayed *AICDA* expression. This indicated uncontrolled mitotic progression, leading to their dramatic expansion without proper immune maturation [53]. Additionally, the strong negative correlation between viral UMIs and host ISG scores identifies AG *MKI67*^+^ PB1 cells as a major viral reservoir, driven by SFTSV-induced suppression of antiviral responses. This could explain the higher viral loads observed in AG ferrets compared to YG. Microscopic analysis further demonstrated disrupted development of BCL6^+^ germinal centers in AG ferrets, accompanied by significant SFTSV presence. This disruption impaired antigen presentation and antibody affinity maturation, contributing to the emergence of dysfunctional and immature plasmablasts. Collectively, these findings suggest that age-related defects in early B cell responses, including the aberrant activation of *T-bet*^+^ ABCs, drive the emergence of dysfunctional plasmablasts in AG ferrets.

While these observations provide valuable mechanistic insight into age-associated B cell dysfunction during SFTSV infection, it is important to recognize that aspects of GC architecture and B cell differentiation may differ between ferrets and humans. Variations in GC organization, T follicular helper (Tfh) cell interactions, and cytokine environments across species could influence plasmablast fate decisions. Therefore, although our results establish key immune mechanisms underlying age-related susceptibility in the ferret model, caution is warranted in directly extrapolating these findings to human disease, and further validation in human systems will be essential.

Comparative scRNA-seq analysis of ferret and human tissues revealed that the *MKI67*^+^ PB1 subset comprised the majority of SFTSV^+^ cells in both ferret groups and human fatal cases, with the highest per-cell viral UMI counts, highlighting its role as a major target for SFTSV infection and replication. Meanwhile, monocytes and MΦs in AG ferrets and human fatal cases displayed low viral UMI counts and weak correlations between viral UMI per cell and ISG scores. However, they exhibited heightened inflammation-related functions, suggesting a role in driving hyperinflammation rather than serving as primary viral reservoirs. The ISG activation in monocytes may reflect their sensitization to limited viral RNA or minimal viral replication, resulting in robust ISG responses [54]. Previous studies have revealed that the SFTSV nonstructural protein (NSs) suppresses host ISG responses by inhibiting ISRE activation and blocking STAT1/2 nuclear translocation, thereby disrupting interferon signaling and antiviral gene expression [55,56]. Consistently, SFTSV^+^ monocytes in both ferret groups and fatal patients showed significantly lower viral UMI/cell compared to *MKI67*^+^ PB1, warranting further investigation into the role of monocytes in systemic SFTSV infection.

While this study employed the highly pathogenic CB1/2014 strain to maintain consistency with previous studies [11,18,27,30], future investigations using diverse SFTSV genotypes, including less virulent strains, will be necessary to fully elucidate how viral genetic variability influences immune responses and disease progression. A key limitation of this study is the inability to perform in vivo validation of certain immune functions, such as antigen-specific B cell responses, class switching, and cytokine-driven HLH mechanisms. These limitations primarily reflect the lack of species-specific immunological tools in the ferret model. For example, efforts to deplete B cells using anti-CD20 antibodies or to assess IgG subclasses with human isotyping kits were unsuccessful due to limited cross-reactivity. Although qRT-PCR analyses supported the scRNA-seq findings, more comprehensive immunological assays will require the development of ferret-compatible reagents. Our study elucidated key cell populations associated with age-dependent systemic damage from SFTSV infection and their potential underlying mechanisms. Especially in the AG ferrets, aberrantly proliferative *MKI67*^+^ PB1s and *T-bet*^+^ ABCs are primary targets of SFTSV, causing dramatic viral expansions across tissues. Furthermore, aged myeloid cells including monocytes and MΦ, marked by heightened inflammatory gene expressions, contribute to hyper-inflammation. These insights into age-dependent dynamics of SFTSV infection could inform targeted interventions for vulnerable groups and have significant implications for vaccine development against fatal SFTSV infections.

## Materials and methods

### Experimental animals and ethics statement

All animal experiments were approved by the Medical Research Institute, a member of the Laboratory Animal Research Center of Chungbuk National University (LARC) (SFTSV infection study: CBNUA-1508-21-02; vaccine treatment study: CBNUA-1963-22-02), and were conducted in strict accordance and adherence to relevant policies regarding animal handling as mandated under the Guidelines for Animal Use and Care of the Korea Centers for Disease Control and Prevention. Viruses were handled in an enhanced biosafety level 3 containment laboratory approved by the Korean Centers for Disease Control and Prevention (KCDC-14-3-07).

### Ferret infection

For the age-dependent infection study, two groups of young-adults (20–24 months of age, ≤ 2 years; n = 26; YG) and aged ferrets (36–40 months of age, ≥ 3 years; n = 26; AG) were prepared. Both groups were inoculated with 0.5 ml of the SFTSV CB1/2014 strain (10^6.0^ TCID_50_/ml; genotype B) via intramuscular (i.m.) route (e.g., outside of the thighs of both legs) [30]. Blood samples were collected from anesthetized ferrets every other day for various assays and virus titration until 10 days post-infection(dpi). Additionally, for single-cell RNA sequencing, three ferrets from each of the infected YG and AG groups were sacrificed at 2, 4, and 6 dpi. Spleen, blood (PBMC), and BM were collected from individual ferrets using different syringes or scissors to avoid cross-contamination.

### Virus titration

Total RNA was isolated from serum and organ samples using TRIzol reagent (Thermo Fisher Scientific) following the standard manufacturer’s protocol. Complementary DNAs were synthesized through reverse transcription using Superscript III (Invitrogen). To determine viral copy numbers, qRT-PCR was conducted using a SYBR green supermix with a primer set specific to the M segment of SFTSV (SFTS-M-F: 5’-AATTCACATTTGAGGGTAGTT-3’, SFTS-M-R: 5’-TATCCAAGGAGGATGACAATAAT-3’). Copy numbers were calculated by comparing the results to a standard control, as previously described [27].

### RNAscope *in situ* hybridization

SFTSV RNA (NP) was detected using the NP-specific probe (Advanced Cell Diagnotics, 552071) and visualized using the RNAscope 2.5 HD Reagent Kit RED (Advanced Cell Diagnotics, 322360). Organ tissue sections were fixed in 10% neutral-buffered formalin and embedded in paraffin (FFPE), according to the manufacturer’s instructions, followed by counterstaining with Gill’s hematoxylin #1 (Polysciences, 24242-1000). Slides were viewed using Olympus IX 71 (Olympus) microscope with DP controller software (v3.2) to capture images.

### Flow cytometry

Single-cell suspensions were prepared from ferret spleens by mechanical dissociation followed by filtration through 70 μm cell strainers. Red blood cells were lysed using RBC lysis buffer (Thermo Fisher Scientific), and the remaining splenocytes were washed and resuspended in FACS buffer (PBS supplemented with 2% FBS and 2 mM EDTA). Fc receptors were blocked by incubating cells with heat-inactivated normal ferret serum for 10 minutes at 4°C.

For B cell infection analysis, splenocytes from young (YG) and aged (AG) ferrets were infected in vitro with the SFTSV CB1/2014 strain at a multiplicity of infection (MOI) of 0.1 and incubated for the indicated time points. After infection, cells were fixed, permeabilized, and stained intracellularly with fluorescence-conjugated antibodies against CD79a (a B cell marker) and SFTSV nucleoprotein (NP). NP⁺ infected cells were quantified within the CD79a ⁺ B cell population.

For T cell and monocyte infection analysis, CD4⁺ and CD8 ⁺ T cells were isolated from splenocytes using a FACSAria Fusion cell sorter (BD Biosciences). The sorted cells were infected with SFTSV (MOI 0.1), stained with fluorescence-conjugated antibodies against CD4, CD8 (surface), and SFTSV NP (intracellular), and analyzed to determine NP⁺ cell frequencies within CD4⁺ and CD8 ⁺ populations.

To assess B cell activation and differentiation, splenocytes were stained with panels of antibodies targeting surface markers (CD79a, CD38, CD27, IgG, IgM, IgA, CD11b, and CD11c) and intracellular markers (Ki-67 and T-bet). Intracellular staining was performed according to the manufacturer’s instructions using appropriate fixation and permeabilization reagents.

Flow cytometry data were acquired using a FACSymphony A3 cytometer (BD Biosciences) and analyzed with FlowJo v10.8 software (BD Biosciences). Dead cells and debris were excluded based on FSC/SSC gating and staining with Fixable Viability Dye (Thermo Fisher Scientific).

### Microneutralization assay

To assess the neutralizing antibody activity against SFTSV in antisera, the focus reduction neutralization test (FRNT_50_) was employed. For the determination of FRNT_50_ values in sera from immunized ferrets, serial twofold dilutions of the sera were mixed with 100 focus-forming units of the SFTSV CB1/2014 strain (genotype B) and incubated for 1 hour. Subsequently, the mixture was used to inoculate confluent Vero E6 cells arranged in 96-well plates, followed by a 1-hour incubation at 37 °C. After removing the inoculum, the cells were replenished with Dulbecco’s Modified Eagle Medium (DMEM) containing 2% FBS and further incubated for 5 days. Following this incubation period, the cells were fixed using 10% neutral-buffered formalin, blocked with 3% BSA, and treated with 10% Triton X-100. The cells were then subjected to incubation with house-generated anti-SFTSV NP antibody, followed by HRP-conjugated anti-mouse IgG antibody. Visualization of foci formation in SFTSV-infected cells was achieved using the 3,3′-diaminobenzidine (DAB) substrate kit from Vector Laboratories. FRNT_50_ values were determined by calculating the reciprocal of the highest dilution at which the number of foci was less than 50% of the number obtained without serum.

### Quantitative real-time PCR (qRT-PCR)

Total RNA was extracted from sorted PB1 and MB1 cells of young (YG) and aged (AG) ferrets infected with SFTSV using the RNeasy Mini Kit (Qiagen) following the manufacturer’s instructions. cDNA synthesis was performed with SuperScript III Reverse Transcriptase (Invitrogen) according to the recommended protocol. Quantitative real-time PCR was carried out using iQ SYBR Green Supermix (Bio-Rad) on a CFX Opus Real-Time PCR System (Bio-Rad). The cycling conditions were as follows: initial denaturation at 95°C for 3 min 30 s, followed by 45 cycles of 95°C for 15 s, 58°C for 15 s and 72°C for 20 s.

Gene-specific primers targeting ferret genes encoding MKI67, CENPF, TOP2A, CDC20, AURKB, STAT4, TACI, BCMA, PPP3CB, TLR7, MyD88, IL-1β, and IL-18 were designed based on ferret sequences available from the NCBI database. GAPDH was used as internal control for normalization. Relative gene expression levels were calculated using the 2^ΔΔCt^ method, and fold-change was normalized to control groups. Statistical significance between groups was assessed using two-way ANOVA. Results are presented as mean ± SEM from at least three independent experiments.

### ELISpot assay

Splenocytes were isolated from ferret spleens by mechanical dissociation using a gentleMACS tissue dissociator (Miltenyi Biotec), followed by filtration through a 70 μm nylon cell strainer to generate single-cell suspensions. Cells were plated at a density of 1 × 10^6^ cells per well in 96-well ELISpot plates (Millipore). To assess antigen-specific cytokine responses, cells were stimulated with either inactivated SFTSV or culture media alone (negative control) for 20–24 hours at 37°C in a 5% CO_2_ incubator. Two ELISpot kits were used for cytokine detection: a ferret-specific IFN-γ ELISpot kit using the monoclonal antibody MTF14 (MabTech), and a cross-reactive IL-2 ELISpot kit based on the porcine IL-2 monoclonal antibody MT265 (MabTech). All procedures were performed according to the manufacturers’ instructions. After the final colorimetric development step, spot-forming units (SFUs), corresponding to individual cytokine-secreting cells, were visualized and counted using an automated ELISpot reader (AID GmbH). Antigen-specific responses were determined by subtracting the number of spots in control wells (media only) from those in virus-stimulated wells.

### Multiplex immunohistochemistry (IHC) staining

Multiplex immunohistochemistry (IHC) was performed for the spleens obtained from control and infected YG and AG ferrets using the Opal-7-color kit (Akoya Biosciences, NEL821001KT), respectively. The tissues were initially fixed with 10% neutral-buffered formalin (NBF) and subsequently embedded in paraffin. Then, these FFPE spleen tissues were sectioned (4 μm) and stored at 20°C until further use. The multiplex IHC procedure, optimized as described previously [57], utilized the Opal IHC Kit (PerkinElmer) in the BOND RX Fully Automated Research Stainer (Leica). Briefly, the staining process was performed using the Leica system, where slides were incubated for 30 minutes at 60°C before deparaffinization with Leica Bioscience Dewax solution (Leica Bioscience, AR9222). Bond Epitope Retrieval 2 (Leica Bioscience, AR9640) was applied for 20 minutes at 100°C. The reacted slides were incubated with Blocking/Ab Diluent for 30 minutes, followed by a one-hour incubation with the primary antibodies including CD20 (Sino biological, 60004-R071), MUM1 (Abcam, AB133590), BCL6 (Abcam, AB172610), SFTSV NP (in house-made), and CD3 (DAKO, M7254), respectively. Opal Polymer HRP Ms + Rb was then applied for 30 minutes, followed by an additional 10-minute incubation with Opal Fluorophore. To eliminate residual antibodies, Bond Epitope Retrieval 2 (Leica Bioscience, AR9640) was applied for 20 minutes, and this process was repeated. Following these steps, DAPI staining was performed for 5 minutes.

### Multiplex IHC imaging and Qupath analysis

We counted the number of SFTSV-infected cells and the germinal center areas in spleen specimens. The stained slides of spleen samples were imaged at 20 × magnification using the PhenoCycler-Fusion system (Akoya Biosciences). Five regions of interest (ROIs) were selected per tissue slide using Qupath software (v0.5.1) [58], specifically centered within or around the follicular regions. Staining quantification was performed by classifying objects and counting SFTSV-positive cells within the ROIs. To measure germinal center areas, we identified regions marked by BCL6^+^ cells. Data from the Qupath analysis were visualized using GraphPad Prism.

### Quantification of hemophagocytosis

The tissues were obtained from SFTSV-infected ferrets at a time point, then dipped in 10% neutral buffered formalin and embedded in paraffin. These FFPE tissues were sectioned in 4-μm sections by microtome and stained with hematoxylin and eosin (H&E). Hemophagocytosis was estimated by counting the numbers of inflammatory MΦ in five randomly selected high-power fields (HPFs). The graphs generated from HLH-counted data were visualized using GraphPad Prism.

### Library preparation for single-cell RNA sequencing

The single-cell RNA libraries were prepared utilizing the 10x Genomics Chromium Single Cell 3′ GEM, Library & Gel Bead kit v3.1 (10X Genomics, PN-1000121) based on the manufacturer’s instructions. Briefly, for each tissue sample, approximately 8,000 cells were loaded in one channel. In each droplet, cDNA was generated through reverse transcription. The quality of the resulting cDNA libraries was evaluated using the TapeStation (Agilent) and quantified with the KAPA library quantification kit (Kapa Biosystems) in accordance with the manufacturer’s library quantification protocol. After cluster amplification of denatured templates, sequencing was performed in a paired-end fashion using the Illumina NovaSeq 6000 platform (Illumina).

### Single-cell RNA sequencing (scRNA-seq) data pre-processing

The Ferret genome (MusPutFur1.0; accession number: GCF_000215625.1) and SFTSV sequences (CB1; accession number: KY789433, KY789436, and KY789439) with related gene annotation and structure files (GTF file) were retrieved from the ENSEMBL database (https://asia.ensembl.org/index.html) and the NCBI nucleotide database (https://www.ncbi.nlm. nih.gov/nucleotide/), respectively (as of November, 2021). With these reference genome sequences, scRNA-seq data obtained from ferret individuals were aligned and quantified using the CellRanger (count algorithm) from 10X Genomics (v6.1.2). The resulting cell-feature count matrix was analyzed by Seurat R packaging (v4.0.6) [59]. To enhance data quality, cell-free RNAs were estimated and removed from the count matrices using the SoupX software (v1.5.0) with the default parameters [60]. Quality control process of scRNA-seq data was performed as previously described, with some modifications [19,61]. Briefly, in each gene-to-cell matrix, individual cells with UMIs count <400 or the number of genes <200 or >8,000 were removed. Additionally, we removed the cells having ≥20% of UMIs mapped to mitochondrial genes. Moreover, doublet cells were identified to investigate virally infected cells being phagocytosed (e.g., SFTSV-infected platelets in MΦ) by using the DoubletFinder software (v2.0) [62]. Following these quality control processes, a total of 384,951 cells were detected in the spleen (134,705 cells), PBMC (109,653 cells), and BM (140,593 cells), respectively.

### Integration of individual samples and transcriptomic network construction

Qualified data from individual samples in each tissue type were normalized and scaled using a negative binomial model of the SCTransform [63], and this process identified the top 3,000 most variable genes. Via reciprocal principal component analysis (RPCA) of these 3,000 genes, the SCT-transformed matrices were integrated for each tissue type to remove batch effects. Following dimensional reduction of the data by the first 50 principal components, the resulting batch-corrected matrix was used to build a k-nearest neighbor graph and determine clusters using the *FindNeighbors* (k = 20) and *FindClusters* functions, respectively. Such transcriptomic graph was visualized for each tissue type using the UMAP method.

### Cell annotation

To identify cell types, we selected DEGs that are upregulated in each cluster relative to the others using *FindMarker* function in Seurat’s implementation with 0.25 log fold changes and a Bonferroni-adjusted *P* < 0.05 (S1 Table). Then, the cell types were manually determined by comparing the upregulated DEGs in clusters to cell type-specific marker genes retrieved from reference literature. Additionally, cluster annotation was verified using the Cellkb software (v2.6) (https://www.cellkb.com/). Through manual inspection of selected DEGs in each cluster, we annotated major cell types across the spleen (resolution of 1.0), PBMC (1.5), and BM (1.0), respectively.

### Detection of scRNA-seq data with SFTSV viral RNA

Despite the absence of polyadenylated mRNA in most bunyaviruses, a recent study demonstrated that internal polyadenylation sites within L, M, and S segments of SFTSV can be captured by the scRNA-seq, and types of cells that harbor SFTSV RNA (SFTSV^+^ cells) in scRNA-seq data are indeed virally infected [19]. However, in droplet-based scRNA-seq, the presence of viral transcript(s) in a cell does not necessarily indicate infection [61,64]. This is because false positive viral counts may arise from the attachment of viruses to the non-host cell surface, the coexistence of ambient free viruses with cells in the droplet suspension, and/or read misalignment. Thus, to enhance the accuracy of SFTSV detection in scRNA-seq data, we established a viral unique molecular identifier (UMI) count cutoff as previously described [64], with some modifications. Briefly, we used the gene-to-cell matrices of count values that were adjusted by SoupX to remove cell-free ambient RNA (above). Then, at various viral UMI counts ranging from 0.0 to 10.0, the differences in SFTSV tropism were computed by subtracting the percentage fraction of non-host cell types of SFTSV from those of host cell types. To this end, the known SFTSV’s target cells, including B cells, PBs, PCs, T cells, MΦs, monocytes, were considered as host cell types [17–19]. Subsequently, in each tissue type, the number of viral UMI counts that maximizes the differences in percentage fraction of SFTSV^+^ cells between known host cell types and non-host cell types was determined as follows:


$$\text{∆T}\left(\text{U}\right)\text{= }{\text{P}}_{\text{host}}\left(\text{U}\right)\text{- }{\text{P}}_{\text{nonhost}}\left(\text{U}\right)$$

where, at the given viral UMI count (U), $\begin{array}{c} \Delta \text{T}\left(\text{U}\right) \end{array}$ indicates the difference in SFTSV tropism, and ${\text{P}}_{\text{host}}\left(\text{U}\right)$ and ${\text{P}}_{\text{nonhost}}\left(\text{U}\right)$ represent the percentage fractions of SFTSV^+^ host cell types and SFTSV^+^ non-host cell types, respectively.

### Pseudotime-based trajectory analyses

To infer the developmental changes of splenic B cells in non-vaccinated YG and AG ferrets during infection, we conducted the trajectory analyses using Monocle2 (v2.30.1) [65]. The trajectory analysis utilized DEGs in the splenic B cell types/subsets. Briefly, the input was generated from the UMI count matrix in Seurat object using the *as.CellDataSet* function with default parameters. The size factors and dispersion of gene expression were estimated. Normalized data dimensions were reduced based on DDRTree using *reduceDimension* with default parameters. From the resulting trajectories, cells belonging to each cell type/subset were aligned along the pseudo-time with the *orderCells* functions, based on the root state of the immature B cell cluster.

### Gene set enrichment analysis and scoring cells for biological functions

Gene set enrichment analysis (GSEA) was conducted using the FGSEA R package (v1.28.0) to identify enriched gene sets between groups of cells, cell types, and/or cell subsets [66]. To this end, we selected DEGs in Seurat’s implementation with 0.25 log fold changes and a Bonferroni-adjusted *P* < 0.05. Functional annotation for each ranked list of enriched gene sets generated by GSEA was performed based on Gene Ontology Biological Process (GOBP) and/or 50 hallmark gene sets in the GSEA Molecular Signatures Database (MSigDB v7.5.1) [67]. Additionally, to analyze the detailed signaling pathway and upstream regulator analysis, we used the Ingenuity Pathway Analysis (v10113820, Qiagen). For functions related to interferon responses, inflammation, aging, inflammasome, and the complement cascade, the cells were scored by evaluating the combined gene expression associated with each function using the *AddModuleScore*, respectively.

### Human scRNA-seq data analysis

The publicly available human scRNA-seq datasets were retrieved from NCBI Gene Expression Omnibus (GEO) repository (accession number: GSE175499 and GSE149313) [18,19]. According to each paper, SFTSV HBMC16 (KY440775–7) and SFTSV KASJH (KP663731–3) genomes were combined with human reference genome (GRCh38) and used for alignment, respectively. The same strategies used for preprocessing, integration, cell annotation, viral RNA detection, and cell composition comparison in the ferret dataset (above) were applied to the analysis. To robustly analyze, the RS group (surviving patients at the recovery phase of disease), AS group (surviving patients at the acute phase of disease), and AD group (deceased patients at the acute phase of disease) from the GSE175499 dataset were incorporated into the Recovery, Infection, and Fatal groups of the GSE149313 dataset, respectively.

### Quantification and statistical analysis

Differences in mean values between two sample groups were computed using two-tailed Wilcoxon rank sum (Mann-Whitney U) test, two-way ANOVA followed by Tukey’s HSD post-test, Kruskal-Wallis test, or Kolmogorov-Smirnov test. All statistical tests were run using custom script using GraphPad Prism (v9.3.1) or R (v3.6.3). Statistical details of our experiments are described in each figure legend. *P* values below 0.05 were considered as statistically significant.

## Supporting information

S1 Fig Age-related phenotypical outcomes and cell type dynamics during SFTSV infection. (TIF)

S2 Fig SFTSV+ cells across three tissues and distinct B cell response in different aged groups. (TIF)

S3 Fig Generation of distinct plasmablast in the aged group during SFTSV infection. (TIF)

S4 Fig Subtyping of B cells, T cells, MΦ, and monocytes, and expression of cell death related genes. (TIF)

S5 Fig Analysis of age-related infection dynamics in ferret BM and PBMC with human PBMC. (TIF)

S6 Fig Expression of genes-related to functional immune response in the spleen and BM across different age groups during SFTSV infection. (TIF)

S1 Table List of differentially expressed genes (DEGs) used for celltype annotation. The marker genes-related to each cell type are highlighted in yellow.
(XLSX)

S2 Table Population of total cells in each celltype and subset. (XLSX)

S3 Table Population of virus+ cells in each celltype and subset. (XLSX)

S1 Data Excel spreadsheet of raw data used to generate Fig 1B, containing aging-related scores in control (ctrl) and infected (inf) cells across three different tissues. (XLSX)
