# Supplementary figures and images for: Age-associated immune dysregulation and B cell dysfunction drive severe outcomes in SFTSV infection
Source: PLoS Pathog. 2025 Aug 12;21(8):e1013402. doi: 10.1371/journal.ppat.1013402 (PMC12342286; doi:10.1371/journal.ppat.1013402)

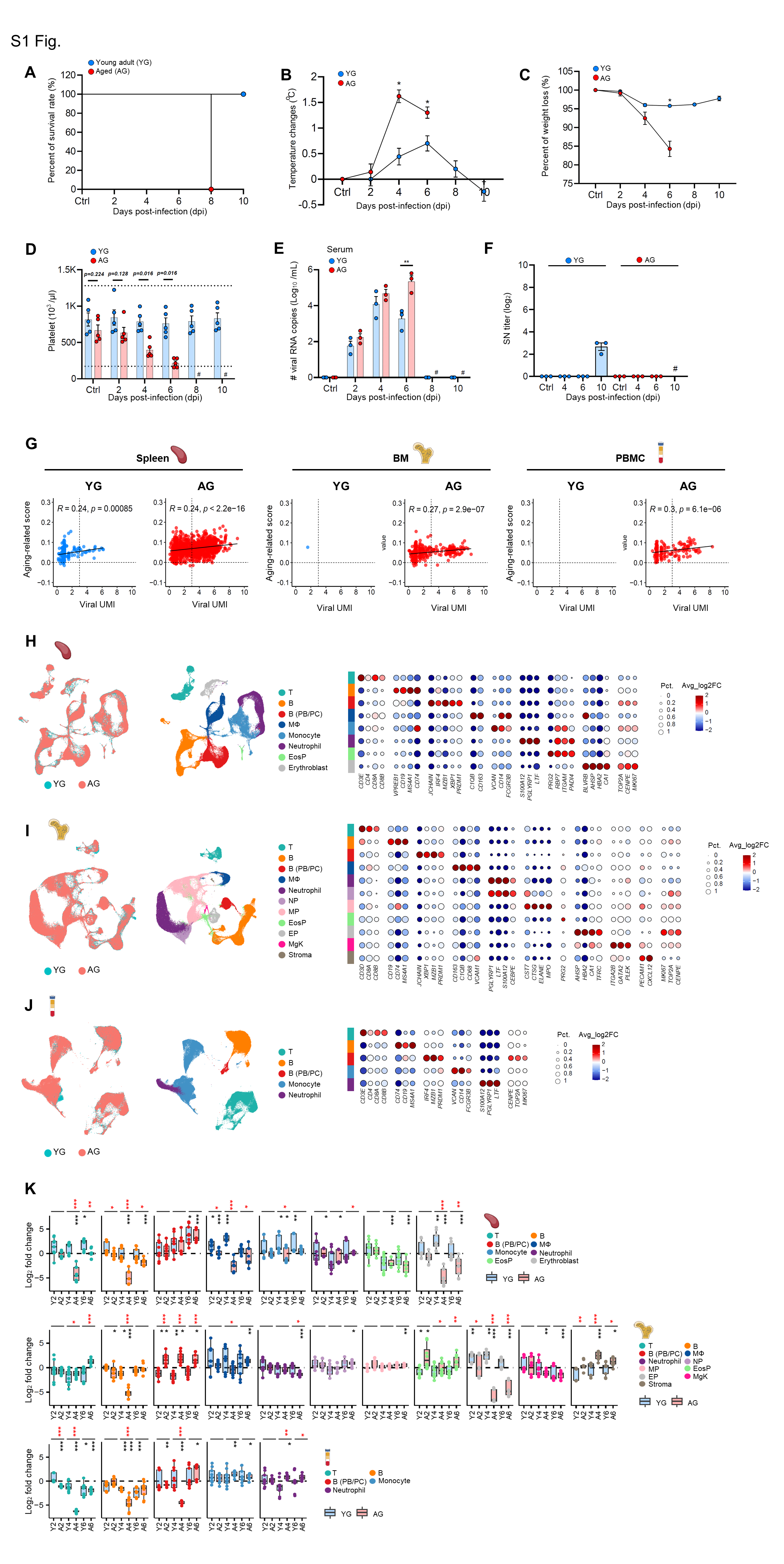

Supplement: S1 Fig — (TIF) [file ppat.1013402.s001.TIF]

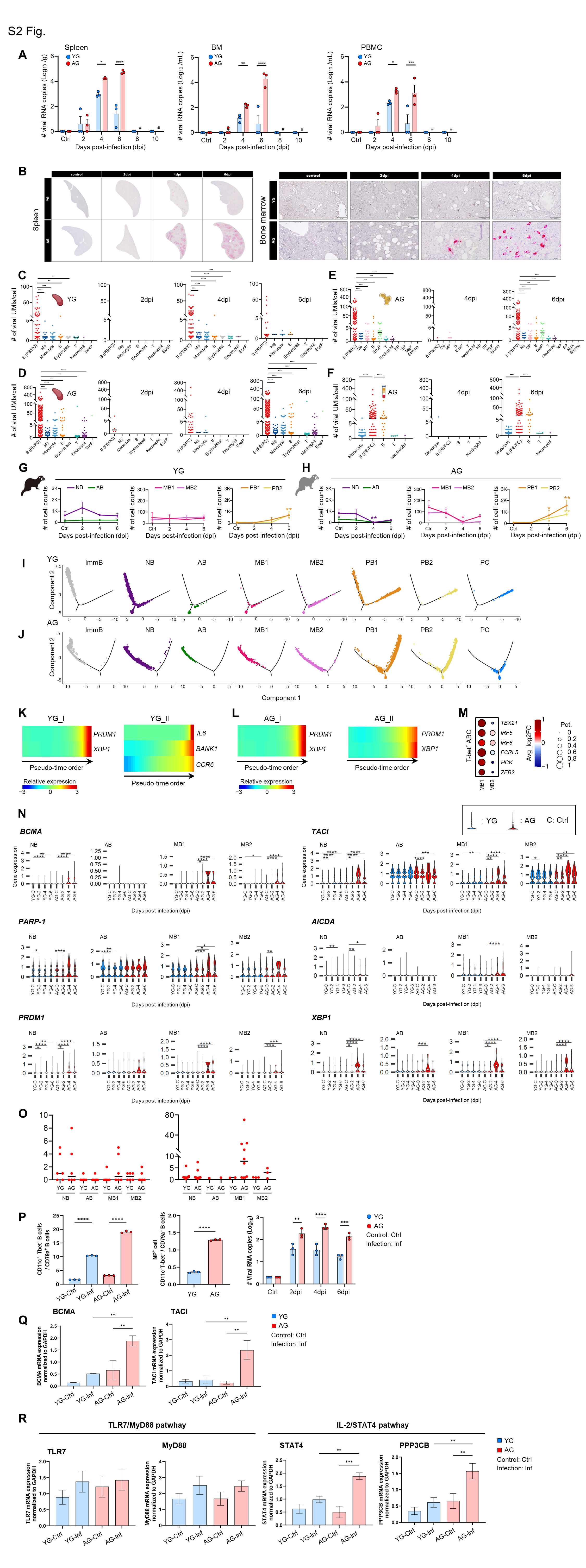

Supplement: S2 Fig — (TIF) [file ppat.1013402.s002.TIF]

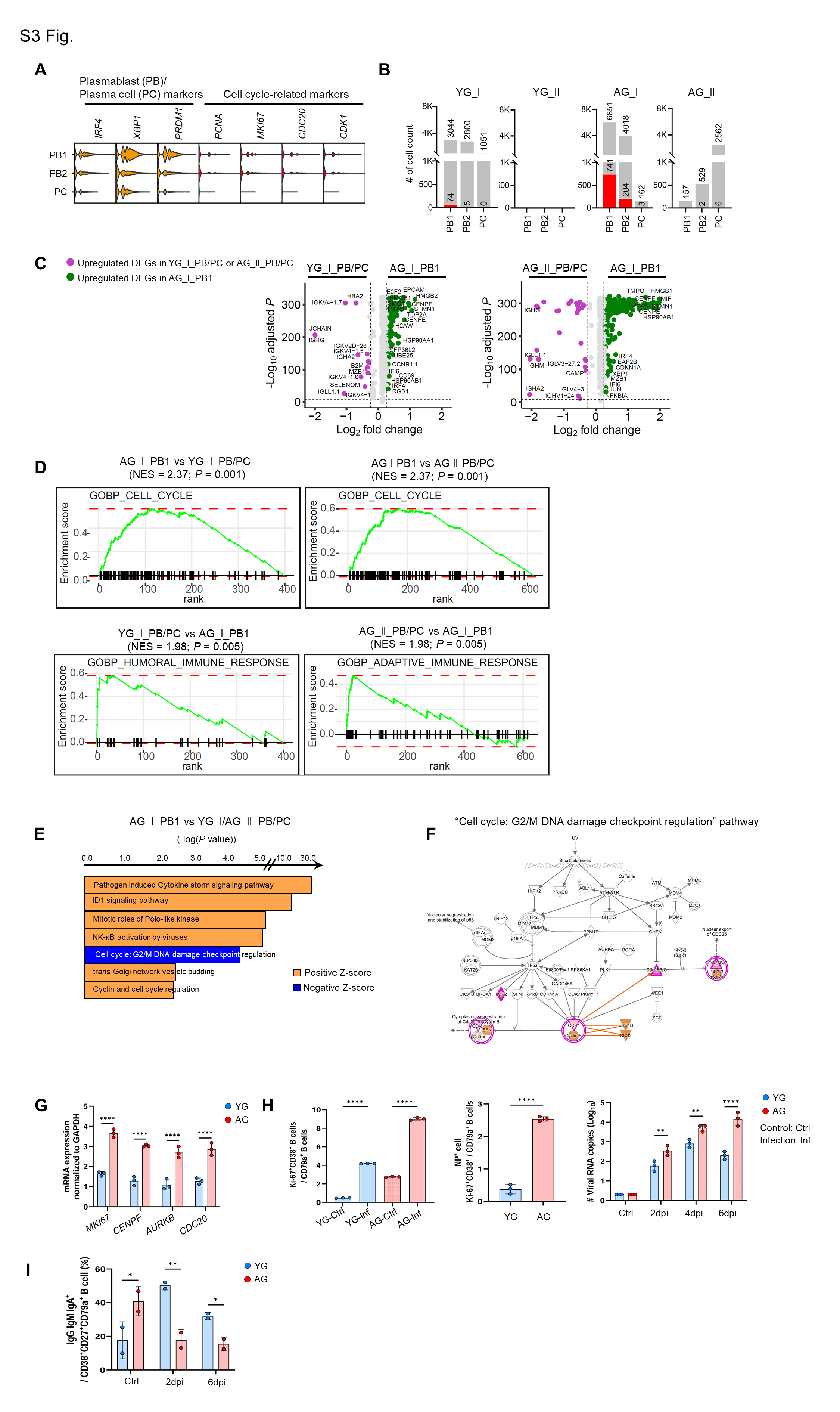

Supplement: S3 Fig — (TIF) [file ppat.1013402.s003.TIF]

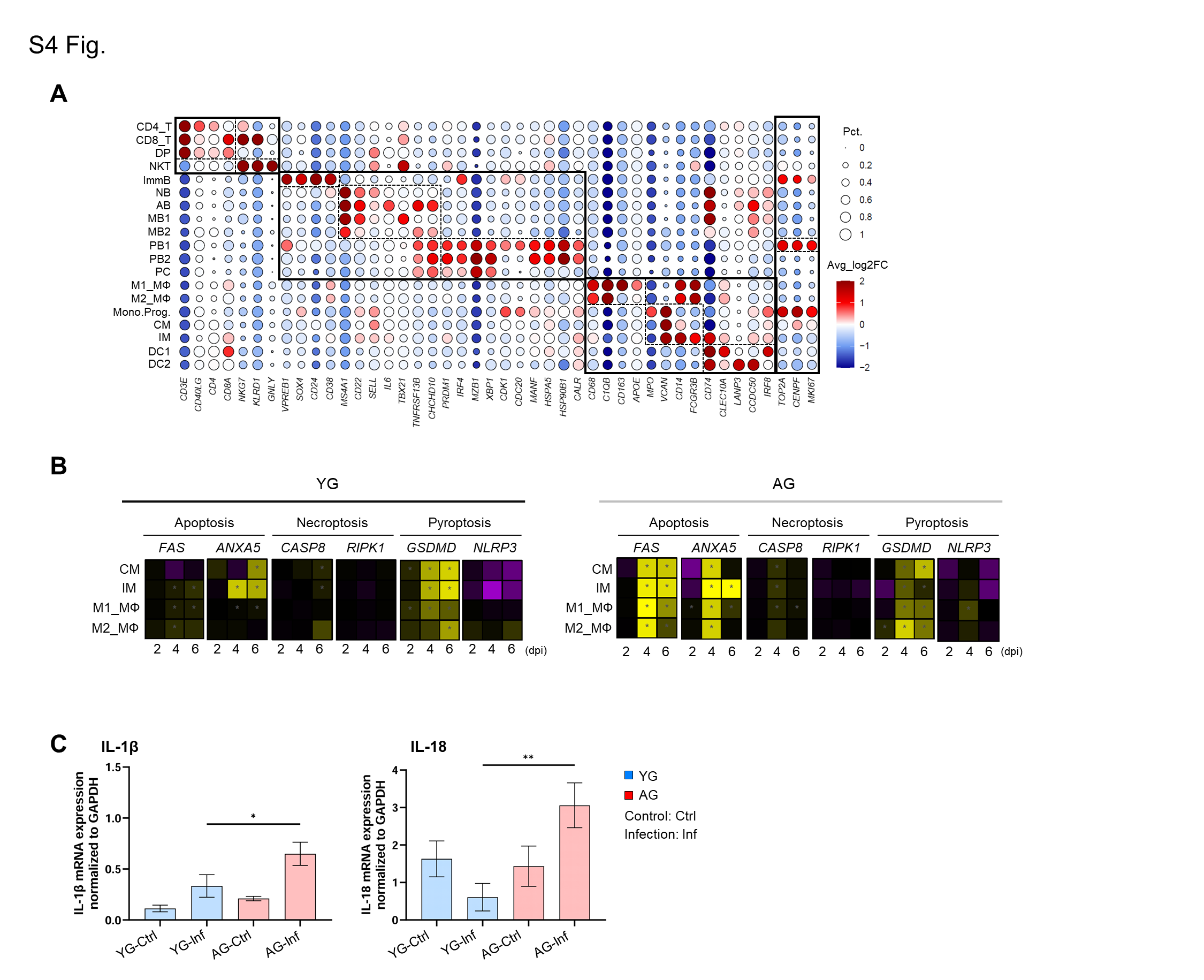

Supplement: S4 Fig — (TIF) [file ppat.1013402.s004.TIF]

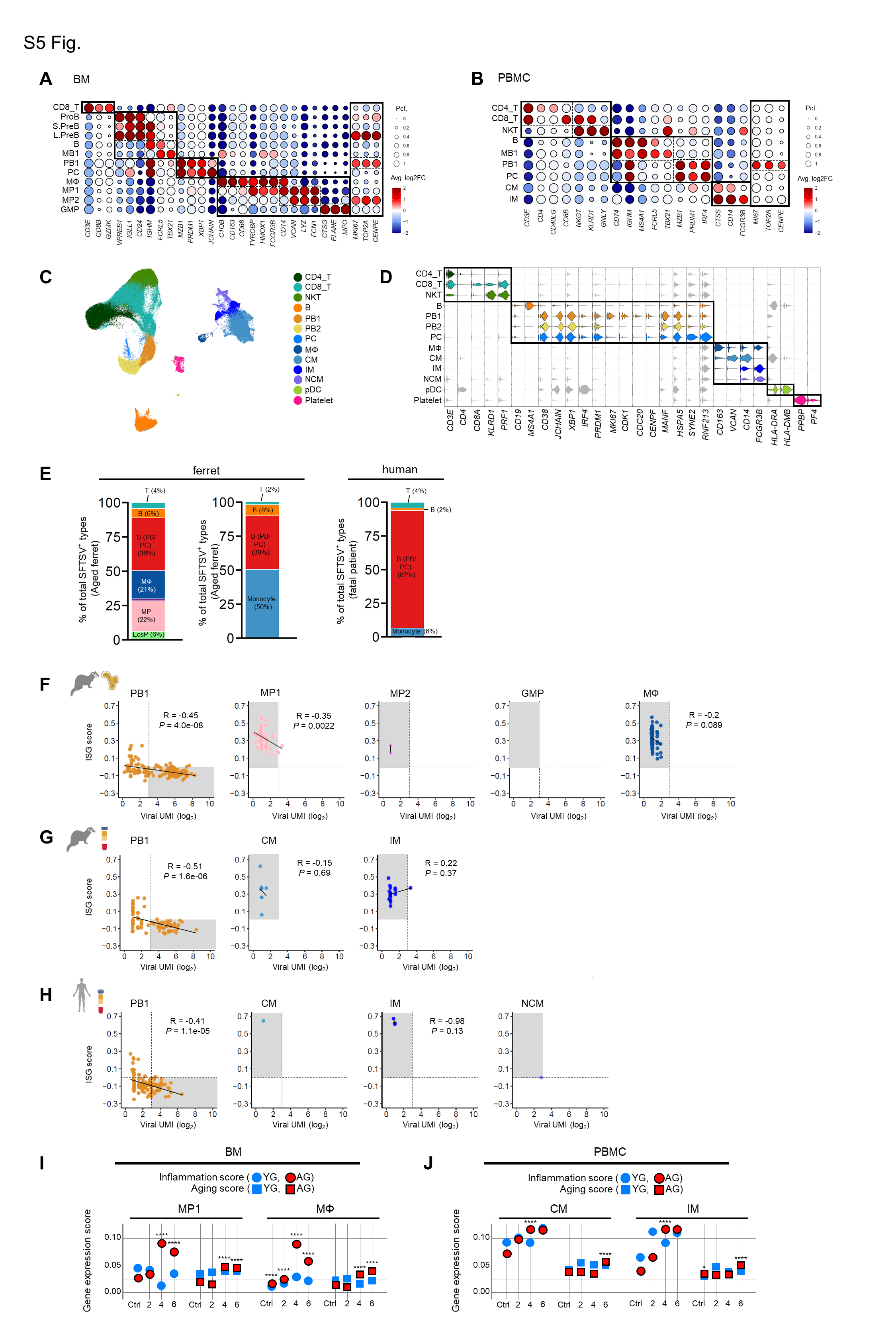

Supplement: S5 Fig — (TIF) [file ppat.1013402.s005.TIF]

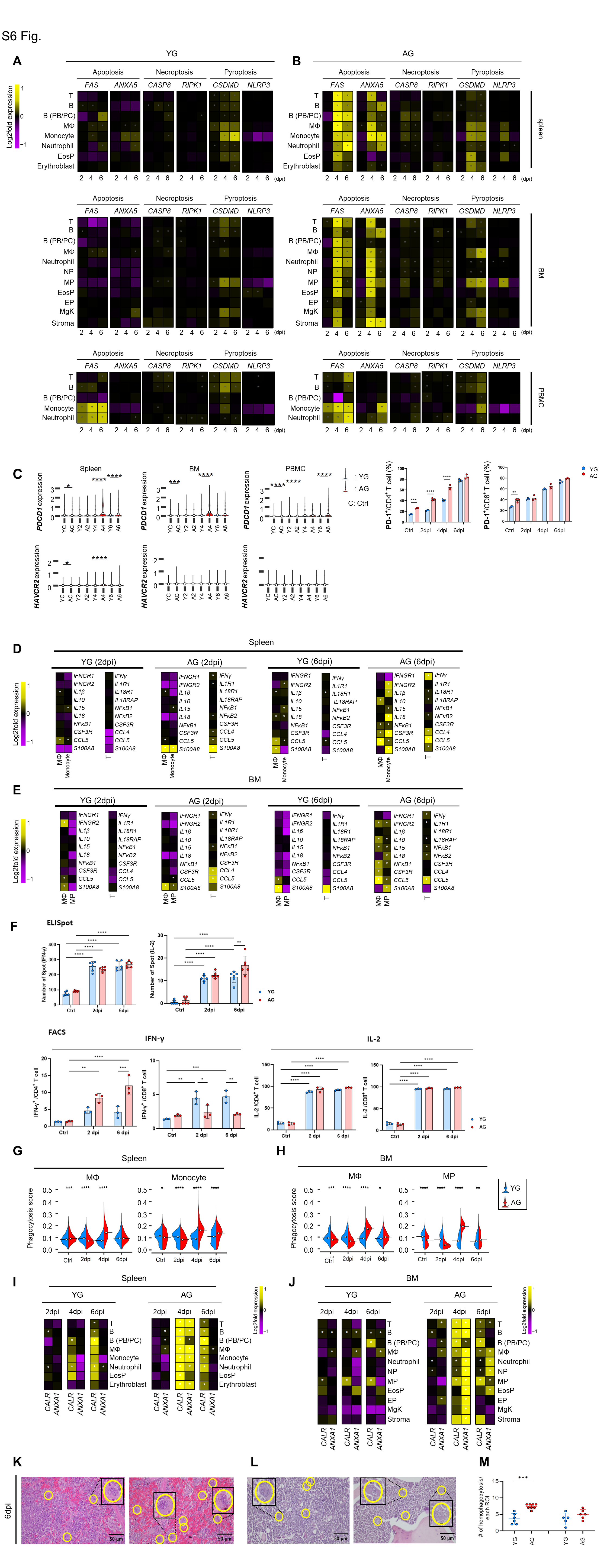

Supplement: S6 Fig — (TIF) [file ppat.1013402.s006.TIF]
